# A road adhesion coefficient-tire cornering stiffness normalization method combining a fractional-order multi-variable gray model with a LSTM network and vehicle direct yaw-moment robust control

**DOI:** 10.3389/fnbot.2023.1229808

**Published:** 2023-08-09

**Authors:** Yufeng Lian, Wenhuan Feng, Shuaishi Liu, Zhigen Nie

**Affiliations:** ^1^School of Electrical and Electronic Engineering, Changchun University of Technology, Changchun, Jilin, China; ^2^Institute of Robotics and Engineering, Changchun University of Technology, Changchun, Jilin, China; ^3^Faculty of Transportation Engineering, Kunming University of Science and Technology, Kunming, Yunnan, China

**Keywords:** direct yaw-moment control, fractional-order multi-variable gray model, LSTM network, normalization method, road adhesion coefficient, tire cornering stiffness

## Abstract

A normalization method of road adhesion coefficient and tire cornering stiffness is proposed to provide the significant information for vehicle direct yaw-moment control (DYC) system design. This method is carried out based on a fractional-order multi-variable gray model (FOMVGM) and a long short-term memory (LSTM) network. A FOMVGM is used to generate training data and testing data for LSTM network, and LSTM network is employed to predict tire cornering stiffness with road adhesion coefficient. In addition to that, tire cornering stiffness represented by road adhesion coefficient can be used to built vehicle lateral dynamic model and participate in DYC robust controller design. Simulations under different driving cycles are carried out to demonstrate the feasibility and effectiveness of the proposed normalization method of road adhesion coefficient and tire cornering stiffness and vehicle DYC robust control system, respectively.

## 1. Introduction

With the development of intelligent transportation technology, people are paying more attention to the acquisition and application of vehicle state and road condition information, which is significant to improve the vehicle adaptability and safety in the different driving environments (Ding et al., 2020). Road adhesion coefficient (Wu et al., 2021; Lian et al., 2022) and tire cornering stiffness (Han et al., 2018; Wang et al., 2022) are generally employed to represent road conditions. It is significant to obtain the information on road adhesion coefficient and tire cornering stiffness for vehicle driving safety.

### 1.1. Road adhesion coefficient identification

**Figure 1:**
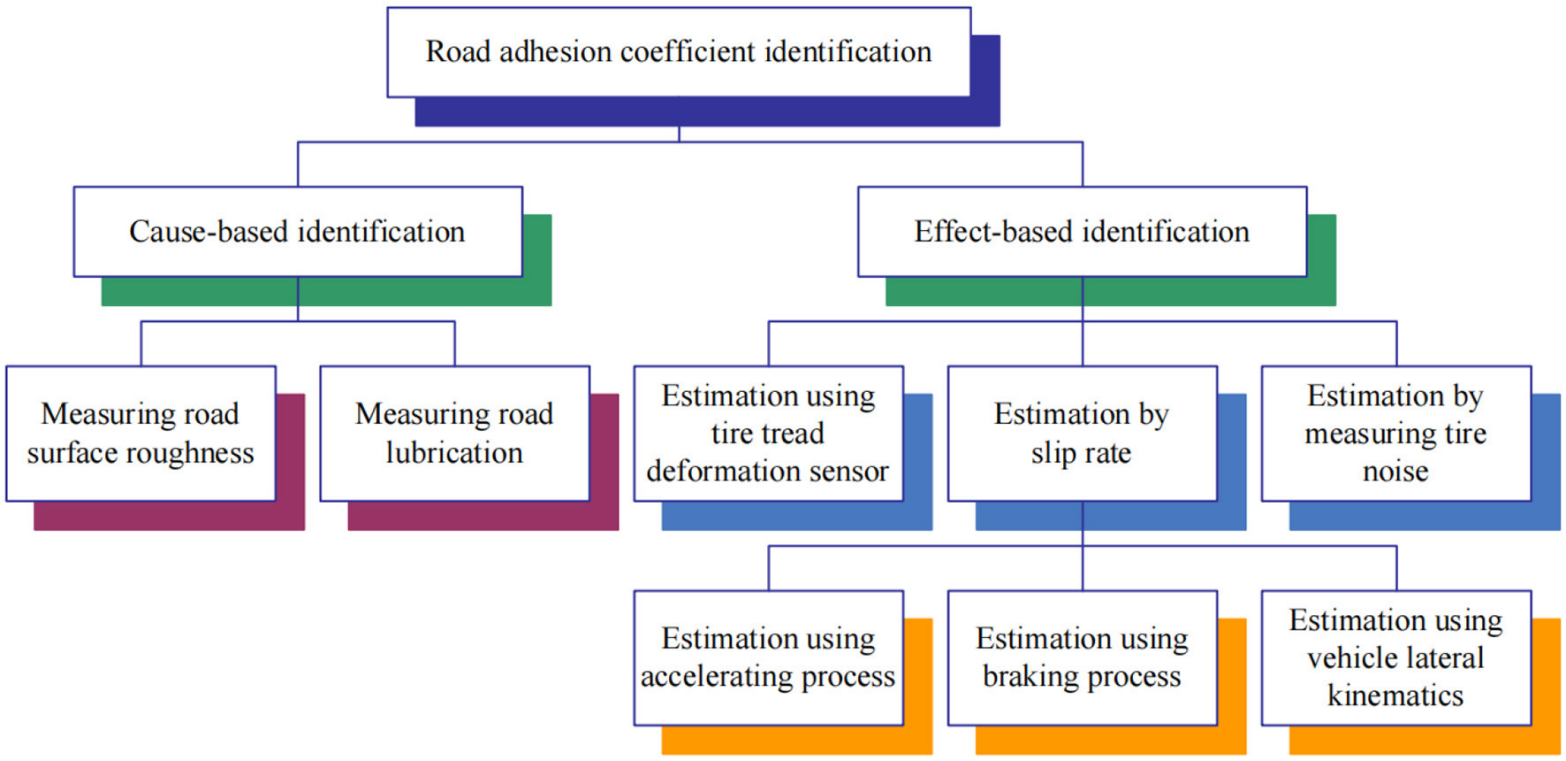
Classification of estimation methods for road adhesion coefficient.

Road adhesion coefficient, which can be represented by the maximum friction coefficient between tire and road surface, is directly related to the vehicle's driving, braking performance, and handling stability. For the identification and acquisition of road adhesion coefficient information, it can perceive the changes of road conditions but also provide necessary information for vehicle control strategy. As shown in Figure 1, the identification methods can be divided into two categories (Müller et al., 2003; Zhang et al., 2022): cause-based identification method and effect-based identification method.

#### 1.1.1. Cause-based identification method

Cause-based identification method can estimate road adhesion coefficient by measuring road factors with optical sensors. A laser line recognition method, which can smooth the texture of road surface and hold the laser line and edges clearly, is proposed with an anisotropic diffusion PM filter for road roughness measurement (Yuan et al., 2015). Android smartphone sensor data are employed to estimate road surface roughness condition by a simple model (Douangphachanh and Oneyama, 2014). This type of identification method requires the installation of sensors. The high cost of sensors make them difficult to commercialize products. Furthermore, this type of identification method needs a lot of test training data, and identification accuracy is mainly affected by experience. Therefore, the low identification accuracy is the main shortcoming in this type of identification method.

#### 1.1.2. Effect-based identification method

Effect-based identification method can estimate road adhesion coefficient by measuring tire factors. Deformation sensor is used to measure tire local strain and strain change to calculate road adhesion coefficient (Breuer et al., 1992). This type of sensor requires energy self-sufficiency and wireless data transmission; therefore, it is expensive and difficult to realize commercial applications. Acoustic sensor is employed to measure the tire/road noise to calculate road adhesion coefficient (Alonso et al., 2015). In addition to that, the μ−*S* curve slope method is one of the most widely applied methods in the effect-based identification method. With the information on wheel speed and vehicle speed, the slip rate of wheel is obtained. The μ−*S* curve slope can be calculated by the least square method or Kalman filter; furthermore, road adhesion coefficient can be calculated (Lee et al., 2004). Road adhesion coefficient can be estimated based on accelerating process (Hahn et al., 2002), braking process (Han et al., 2017), and vehicle lateral kinematics (Junmin et al., 2004). In this type of identification method, the detection information is affected by tire type, tire pressure, tire wear degree, and other factors, and the noise is larger.

### 1.2. Tire cornering stiffness estimation

Tire cornering stiffness is one of the vehicle model parameters, which can also describe road condition, especially, vehicle lateral dynamics system modeling and vehicle controller design. Tire cornering stiffness can be changed resulting from road friction and tire slip angle. Therefore, it is hard to obtain directly tire cornering stiffness data resulting from the non-linear characteristics of tire principally. Numerous estimation methods have been developed to acquire tire cornering stiffness data (Han et al., 2018). On one hand, tire cornering stiffness can participate in vehicle parameter estimation, such as vehicle sideslip angle (Lian et al., 2015). On the other hand, it can be also involved in the design of the active steering control system, such as vehicle direct yaw-moment control (DYC) system (Lian et al., 2018). The estimation methods of tire cornering stiffness can be divided into two groups mainly; one group is simultaneous estimation of tire cornering stiffness with anther vehicle states, such as sideslip angle. A fuzzy adaptive robust cubature Kalman filter is designed to estimate sideslip angle and tire cornering stiffness, and recursive least squares (RLS) is employed to update filter parameters (Wang et al., 2022). For a in-wheel motor drive electric vehicle, the adaptive capability of driving cycle, which is becoming more and more significant in modern transportation, needs to be improved in trajectory tracking procedure. Tire cornering stiffness is regarded as a time-varying parameter, which can be modified by model predictive control (Li and Yang, 2022). Another group is single estimation of tire cornering stiffness without other vehicle states. Tire lateral force sensor is developed and provides a new solution for estimating tire cornering stiffness both in academic research and industry circles (Nam et al., 2012). Lateral force information from lateral force sensors is used in regression model of the RLS method with forgetting factors and constraints (Lian et al., 2015). An unscented Kalman filter is used to estimate sideslip angle, and a forgetting factor RLS method is used to estimate tire cornering stiffness (Tian et al., 2019). These methods mentioned above make estimator burden in computational process resulting in complication, besides noise and various sensor installation and environmental constraints.

The main contributions of this study are as follows:

(1) A road adhesion coefficient-tire cornering stiffness normalization method combining a fractional-order multi-variable Gray model (FOMVGM) with a long short-term memory (LSTM) network is proposed. Tire cornering stiffness can be represented by road adhesion coefficient, and tire cornering stiffness estimation algorithm can be omitted to reduce the computation burden of the tire cornering stiffness estimation.

(2) A semi-uncertainty dynamic model (SUDM), which is based on a vehicle lateral dynamic model with road adhesion coefficient, is proposed. It can ensure the vehicle's handling stability and restrain the influence of uncertain factors caused by parameter perturbation and external environment interference but also make the single-variable yaw rate control instead of two-variable control of yaw rate and sideslip angle.

The remaining study is organized as follows. In Section 2, a FOMVGM and a LSTM network-based normalization method is proposed and described in details. In Section 3, an SUDM with road adhesion coefficient is proposed, and a direct yaw-moment robust controller based on SUDM is presented. Simulation experimental results and analysis are presented in Section 4. Conclusions are summarized in Section 5.

## 2. A normalization with a FOMVGM and a LSTM network

**Figure 2:**
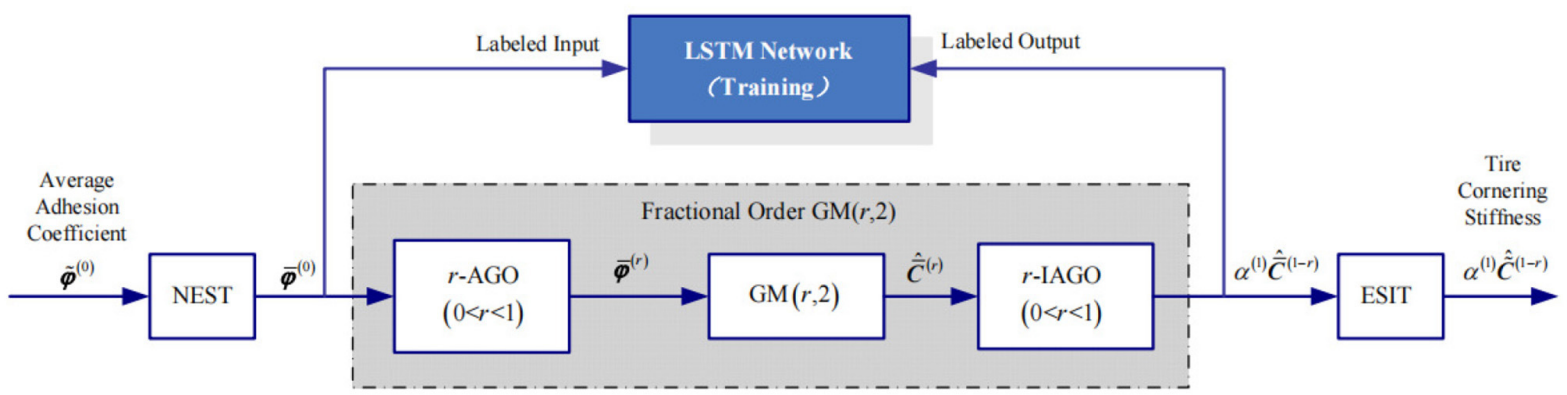
The structure diagram of a FOMVGM and LSTM-based normalization method.

A normalization method of road adhesion coefficient and tire cornering stiffness is proposed, and the structure diagram is shown in Figure 2. A LSTM network is used to normalize between road adhesion coefficient and tire cornering stiffness. There is not enough original data to train and test LSTM network for road adhesion coefficient and tire cornering stiffness. Therefore, a FOMVGM, which can deal with small samples, is built to provide numerous data as the training and testing data for LSTM network.

### 2.1. LSTM network-based normalization

**Figure 3:**
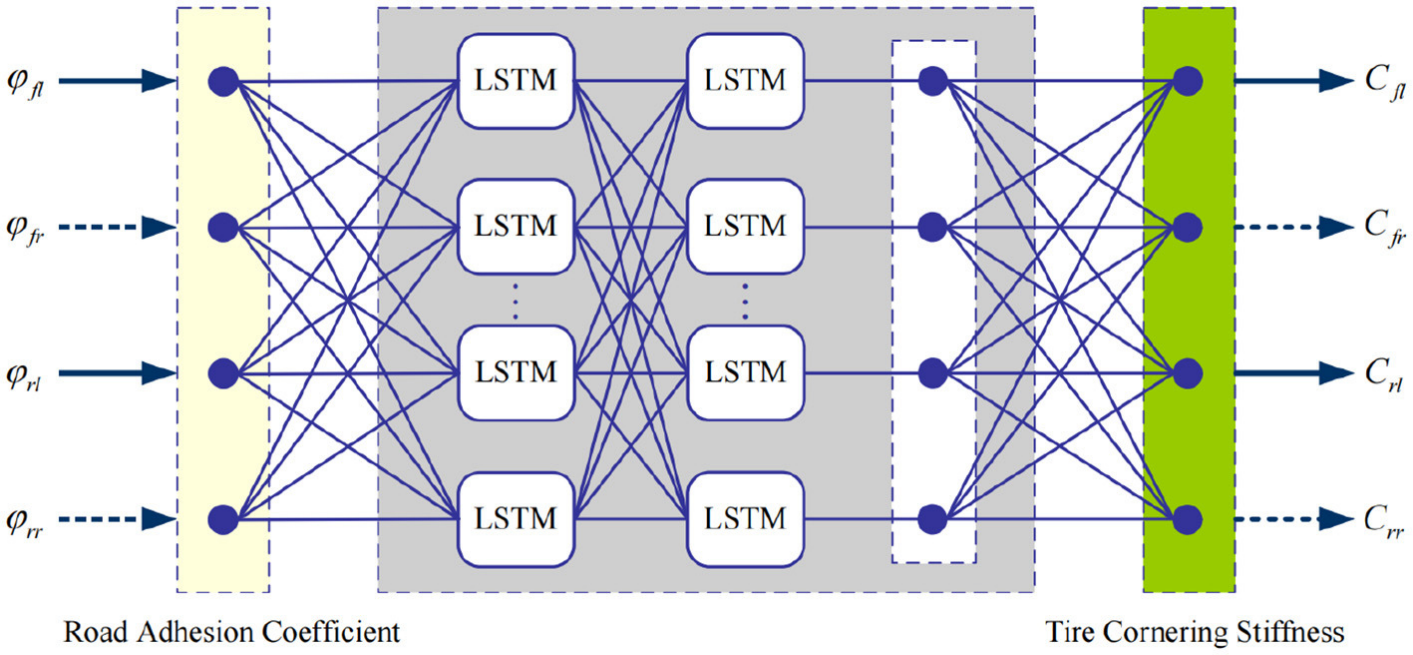
LSTM network structure.

LSTM network is proposed to solve the problem on vanishing gradient. On the basis of recurrent neural network (RNN), each ordinary node in the hidden layer is replaced by a memory cell with input gate, forget gate, and output gate. LSTM network can balance the weight between history input information and current input information through the calculations of input gate, forget gate, and output gate and the update of the memory cell state (Shi et al., 2021). As shown in Figure 3, the regression model of the LSTM network is constructed with an input layer with four nodes, and two LSTM layers employed to extract road adhesion coefficient feature, a full connected layer, and a regression layer with four nodes.

### 2.2. A FOMVGM modeling

Driving intention and average adhesion coefficient sequences are collected at different time intervals, namely, non-equidistant sequences. They are not suitable for directly participating in FOMVGM modeling. Therefore, driving intention and average adhesion coefficient sequences need to be transformed to equidistant sequences before FOMVGM modeling.

#### 2.2.1. Non-equidistant sequence transformation (NEST)

Let ${\tilde{\text{x}}}^{\left(0\right)}=\left[{\tilde{\text{x}}}^{\left(0\right)}\left(t_{1}\right),{\tilde{\text{x}}}^{\left(0\right)}\left(t_{2}\right),\cdots \;,{\tilde{\text{x}}}^{\left(0\right)}\left(t_{n}\right)\right]$ be a non-equidistant sequence, an equidistant sequence can be transformed by (1).


(1) $$\begin{array}{l} {\bar{x}}^{\left(0\right)}=\left[{\bar{\text{x}}}^{\left(0\right)}\left(1\right),{\bar{\text{x}}}^{\left(0\right)}\left(2\right),\cdots \;,{\bar{\text{x}}}^{\left(0\right)}\left(n\right)\right] \end{array}$$

where, $\{\begin{array}{ll} {\bar{x}}^{\left(0\right)}\left(1\right)={\tilde{x}}^{\left(0\right)}\left(t_{1}\right), & i=1\\ {\bar{x}}^{\left(0\right)}\left(i\right)={\tilde{x}}^{\left(0\right)}\left(t_{i}\right)-\text{Δ}{\tilde{x}}^{\left(0\right)}\left(t_{i}\right), & i=2,3,\cdots ,n \end{array}\text{Δ}{\tilde{x}}^{\left(0\right)}\left(t_{i}\right)$, $\Delta {\tilde{\text{x}}}^{\left(0\right)}\left(t_{i}\right)$ is total difference for every interval, and the calculation formulas of $\Delta {\tilde{\text{x}}}^{\left(0\right)}\left(t_{i}\right)=\mu \left(i\right)\left[{\tilde{\text{x}}}^{\left(0\right)}\left(t_{i}\right)-{\tilde{\text{x}}}^{\left(0\right)}\left(t_{i-1}\right)\right],i=2,3,\cdots \;,n$; $\mu \left(i\right)=\frac{t_{i}-\left(i-1\right)\Delta t}{\Delta t},i=2,3,\cdots \;,n$, is the coefficient between $\left[{\tilde{\text{x}}}^{\left(0\right)}\left(t_{i}\right)-{\tilde{\text{x}}}^{\left(0\right)}\left(t_{i-1}\right)\right]$ and $\Delta t=\frac{t_{n}-t_{2}}{n-1},i=2,3,\cdots \;,n$; Δ*t* is average interval; *n* is the number of ${\bar{\text{x}}}^{\left(0\right)}$.

#### 2.2.2. FOMVGM(*r*,2) modeling

**Definition 1**: A sequence ${\bar{\text{x}}}^{\left(r\right)}={\left[{\bar{\text{x}}}^{\left(r\right)}\left(1\right),{\bar{\text{x}}}^{\left(r\right)}\left(2\right),\cdots \;,{\bar{\text{x}}}^{\left(r\right)}\left(n\right)\right]}^{T}$ can be obtained with ${\bar{\text{x}}}^{\left(0\right)}={\left[{\bar{\text{x}}}^{\left(0\right)}\left(1\right),{\bar{\text{x}}}^{\left(0\right)}\left(2\right),\cdots \;,{\bar{\text{x}}}^{\left(0\right)}\left(n\right)\right]}^{T}$ by *r*-order accumulating generation operator (*r*-AGO), namely,


(2) $$\begin{array}{l} {\bar{x}}^{\left(r\right)}=Q\left(r\right){\bar{x}}^{\left(0\right)} \end{array}$$

where, $\text{Q}\left(r\right)={\left[\begin{array}{ccccc} 1 & 0 & 0 & \cdots & 0\\ C_{r}^{1} & 1 & 0 & \cdots & 0\\ C_{r+1}^{2} & C_{r}^{1} & 1 & \cdots & 0\\ \vdots & \vdots & \vdots & \ddots & 0\\ C_{n-1+r-1}^{n-1} & C_{n-1+r-1}^{n-2} & \cdots & C_{r}^{1} & 1 \end{array}\right]}_{n\times n}$; ${\bar{\text{x}}}^{\left(0\right)}\left(k\right)\geq 0$, *k* = 1, 2, ⋯ , *n*; ${\bar{\text{x}}}^{\left(r\right)}$ is the *r*-order accumulated generating sequence of ${\bar{\text{x}}}^{\left(0\right)}$; 0 < *r* < 1; $C_{r-1}^{0}=1$; $C_{k-i+r-1}^{k-i}=\frac{\left(k-i+r-1\right)\left(k-i+r-2\right)\ldots \left(r+1\right)r}{\left(k-i\right)!}$; $C_{k}^{k+1}=0$.

**Definition 2**: A sequence $\alpha^{\left(1\right)}{\bar{\text{x}}}^{\left(1-r\right)}$ can be obtained with ${\bar{\text{x}}}^{\left(0\right)}$ by the *r*-order inverse AGO (*r*-IAGO), namely,


(3) $$\begin{array}{l} \alpha^{\left(1\right)}{\bar{\text{x}}}^{\left(1-r\right)}\left(k\right)={\bar{\text{x}}}^{\left(1-r\right)}\left(k\right)-{\bar{\text{x}}}^{\left(1-r\right)}\left(k-1\right) \end{array}$$

where, $\alpha^{\left(1\right)}{\bar{\text{x}}}^{\left(1-r\right)}={\left[\alpha^{\left(1\right)}{\bar{\text{x}}}^{\left(1-r\right)}\left(1\right),\cdots \;,\alpha^{\left(1\right)}{\bar{\text{x}}}^{\left(1-r\right)}\left(n\right)\right]}^{T}$ is the *r*-order inverse accumulated generating sequence of ${\bar{\text{x}}}^{\left(0\right)}$.

Definition 2 is applied in model reduction. Model values obtained by Gray model calculation are based on *r*-order accumulated generating sequence ${\bar{\text{x}}}^{\left(r\right)}$. They should be used to fit or predict original sequences after *r*-IAGO.

**Definition 3**: Let a system behavior characteristic sequence be ${\bar{\text{x}}}_{1}^{\left(0\right)}=\left[{\bar{\text{x}}}_{1}^{\left(0\right)}\left(1\right),{\bar{\text{x}}}_{1}^{\left(0\right)}\left(2\right),\cdots \;,{\bar{\text{x}}}_{1}^{\left(0\right)}\left(n\right)\right]$, correlation factor sequence be ${\bar{\text{x}}}_{2}^{\left(0\right)}=\left[{\bar{\text{x}}}_{2}^{\left(0\right)}\left(1\right),{\bar{\text{x}}}_{2}^{\left(0\right)}\left(2\right),\cdots \;,{\bar{\text{x}}}_{2}^{\left(0\right)}\left(n\right)\right]$, and adjacent neighbor sequence of ${\bar{\text{x}}}_{1}^{\left(0\right)}$ be ${\bar{\text{z}}}_{1}^{\left(r\right)}=\left[{\bar{\text{z}}}_{1}^{\left(r\right)}\left(2\right),{\bar{\text{z}}}_{1}^{\left(r\right)}\left(3\right),\cdots \;,{\bar{\text{z}}}_{1}^{\left(r\right)}\left(n\right)\right]$. A fractional order multi-variable GM(*r*,2) can be formulated as follows:


(4) $$\begin{array}{l} \alpha^{\left(1\right)}{\bar{\text{x}}}_{1}^{\left(r\right)}\left(k\right)+a{\bar{\text{z}}}_{1}^{\left(r\right)}\left(k\right)=b{\bar{\text{x}}}_{2}^{\left(r\right)}\left(k\right) \end{array}$$

where, ${\bar{\text{z}}}_{1}^{\left(r\right)}\left(k\right)=\frac{1}{2}\left[{\bar{\text{x}}}_{1}^{\left(r\right)}\left(k\right)+{\bar{\text{x}}}_{1}^{\left(r\right)}\left(k-1\right)\right],k=2,3,\cdots \;,n$; ${\bar{\text{x}}}_{1}^{\left(r\right)}\left(k\right)$ and ${\bar{\text{x}}}_{2}^{\left(r\right)}\left(k\right)$ can be calculated by Definition 1.

**Theorem 1**: Least square estimation parameters of $\alpha^{\left(1\right)}{\bar{\text{x}}}_{1}^{\left(r\right)}\left(k\right)+a{\bar{\text{z}}}_{1}^{\left(r\right)}\left(k\right)=b{\bar{\text{x}}}_{2}^{\left(r\right)}\left(k\right)$ can satisfy as follows:


(5) $$\begin{array}{l} \left[\begin{array}{c} a\\ b \end{array}\right]={\left(B^{T}B\right)}^{-1}B^{T}Y \end{array}$$

where, $\text{B}=\left[\begin{array}{cc} -\frac{1}{2}\left[{\bar{\text{x}}}_{1}^{\left(r\right)}\left(2\right)+{\bar{\text{x}}}_{1}^{\left(r\right)}\left(1\right)\right] & {\bar{\text{x}}}_{2}^{\left(r\right)}\left(2\right)\\ -\frac{1}{2}\left[{\bar{\text{x}}}_{1}^{\left(r\right)}\left(3\right)+{\bar{\text{x}}}_{1}^{\left(r\right)}\left(2\right)\right] & {\bar{\text{x}}}_{2}^{\left(r\right)}\left(3\right)\\ \vdots & \vdots \\ -\frac{1}{2}\left[{\bar{\text{x}}}_{1}^{\left(r\right)}\left(n\right)+{\bar{\text{x}}}_{1}^{\left(r\right)}\left(n-1\right)\right] & {\bar{\text{x}}}_{2}^{\left(r\right)}\left(n\right) \end{array}\right]$;

$\text{Y}=\left[\begin{array}{c} {\bar{\text{x}}}_{1}^{\left(r\right)}\left(2\right)-{\bar{\text{x}}}_{1}^{\left(r\right)}\left(1\right)\\ {\bar{\text{x}}}_{1}^{\left(r\right)}\left(3\right)-{\bar{\text{x}}}_{1}^{\left(r\right)}\left(2\right)\\ \vdots \\ {\bar{\text{x}}}_{1}^{\left(r\right)}\left(n\right)-{\bar{\text{x}}}_{1}^{\left(r\right)}\left(n-1\right) \end{array}\right]$.

The model values of GM(*r*,2) can be represented as $\alpha^{\left(1\right)}{\hat{\bar{\text{x}}}}_{1}^{\left(1-r\right)}$, which is derived from ${\hat{\bar{\text{x}}}}_{1}^{\left(r\right)}$ by *r*-IAGO.

#### 2.2.3. Equidistant sequence inverse-transformation (ESIT)

Reduced sequence of FOMVGM is a still equidistant sequence, which is not corresponded to actual driving intention. Therefore, reduced sequence needs to be transformed to non-equidistant sequences ${\hat{\tilde{\text{x}}}}_{1}^{\left(0\right)}$.


(6) $$\begin{array}{l} \{\begin{array}{ll} {\hat{\tilde{x}}}_{1}^{\left(0\right)}\left(t_{1}\right)=\alpha^{\left(1\right)}{\hat{\bar{x}}}_{1}^{\left(1-r\right)}\left(1\right), & j=1\\ {\hat{\tilde{x}}}_{1}^{\left(0\right)}\left(t_{j}\right)=\alpha^{\left(1\right)}{\hat{\bar{x}}}_{1}^{\left(1-r\right)}\left(j\right)+\text{Δ}\alpha^{\left(1\right)}{\hat{\bar{x}}}_{1}^{\left(1-r\right)}\left(t_{j}\right), & others \end{array} \end{array}$$

where, ${\hat{\tilde{\text{x}}}}_{1}^{\left(0\right)}\left(t_{j}\right)$ is the fitting values of GM(*r*,2), *j* = 1, 2, ⋯ , *n*; ${\hat{\tilde{\text{x}}}}_{1}^{\left(0\right)}\left(t_{j}\right)$ is the prediction values of GM(*r*,2), *j* = *n*+1, *n*+2, ⋯ , *n*+*p*; $\Delta \alpha^{\left(1\right)}{\hat{\bar{\text{x}}}}_{1}^{\left(1-r\right)}\left(t_{j}\right)=\bar{\mu }\left(j\right)|{\hat{\tilde{\text{x}}}}_{1}^{\left(0\right)}\left(t_{j}\right)-{\hat{\tilde{\text{x}}}}_{1}^{\left(0\right)}\left(t_{j-1}\right)|,i=2,3,\cdots \;,n+p$, is total difference for every interval; $\bar{\mu }\left(t_{j}\right)=\frac{t_{j}-\left(j-1\right)\Delta \bar{t}}{\Delta \bar{t}},j=2,3,\cdots \;,n+p$, is coefficient between $\left|{\hat{\tilde{\text{x}}}}_{1}^{\left(0\right)}\left(t_{j}\right)-{\hat{\tilde{\text{x}}}}_{1}^{\left(0\right)}\left(t_{j-1}\right)\right|$ and $\Delta \bar{t}$; $\Delta \bar{t}=\frac{1}{n-1}\overset{n}{\underset{i=2}{\sum }}\Delta t_{i}=\frac{t_{n}-t_{2}}{n-1},i=2,3,\cdots \;,n$, is average interval; *p* is the number of prediction values.

## 3. Direct yaw-moment robust control

### 3.1. Vehicle lateral dynamic modeling

**Figure 4:**
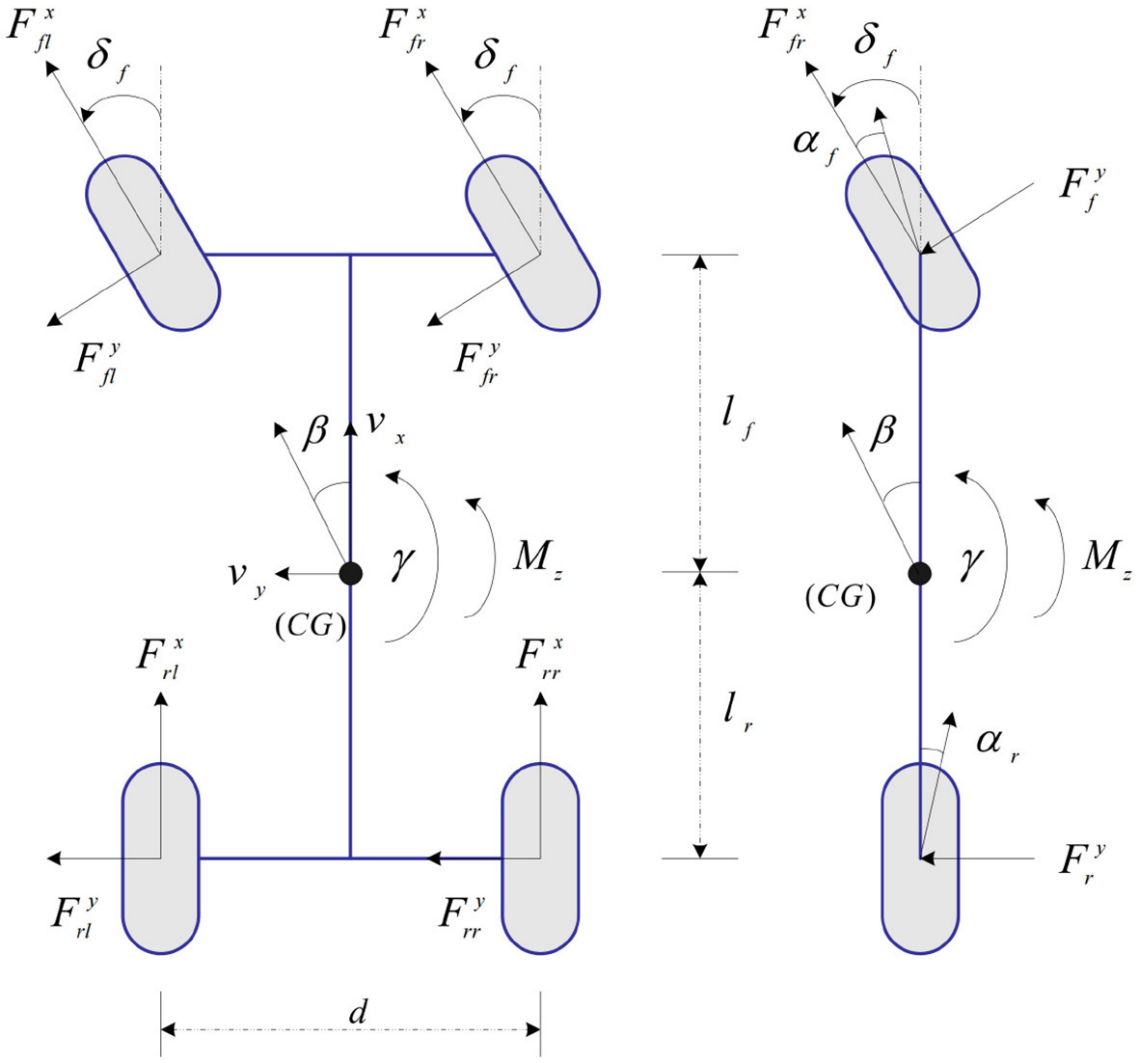
Planar vehicle models: Four-wheel model and two-wheel model.

A 2 degree-of-freedom (DOF) vehicle model in longitudinal and lateral planes is shown in Figure 4. The yaw angle around the vertical axis is taken as positive in the anti-clockwise direction. Vehicle longitudinal direction is represented with *x*, and vehicle lateral direction is represented with *y*. Assuming that φ_*fl*_ = φ_*fr*_ = φ_*f*_, φ_*rl*_ = φ_*rr*_ = φ_*r*_, *C*_*fl*_(φ_*f*_) = *C*_*fr*_(φ_*f*_) = *C*_*f*_(φ_*f*_), and *C*_*rl*_(φ_*r*_) = *C*_*rr*_(φ_*r*_) = *C*_*r*_(φ_*r*_). Figure 3 can be simplified as a two-node input and two-node output LSTM network. Furthermore, the linear motion equations of two-input and one-output are given by Lian et al. (2018),


(7) $$\begin{array}{l} \{\begin{array}{l} \overset{.}{x}=Ax+Bu\\ y=Cx \end{array} \end{array}$$

where, **x** = [β, γ]^*T*^; $\text{u}={\left[\delta_{f},M_{z}\right]}^{T}$; **y** = γ; $\text{A}=\left[\begin{array}{cc} -\frac{2\left(C_{f}\left(\varphi_{f}\right)+C_{r}\left(\varphi_{r}\right)\right)}{mv_{x}} & \frac{2\left(C_{r}\left(\varphi_{r}\right)l_{r}-C_{f}\left(\varphi_{f}\right)l_{f}\right)}{mv_{x}^{2}}-1\\ \frac{2\left(C_{r}\left(\varphi_{r}\right)l_{r}-C_{f}\left(\varphi_{f}\right)l_{f}\right)}{I_{z}} & -\frac{2\left(C_{f}\left(\varphi_{f}\right)l_{f}^{2}+C_{r}\left(\varphi_{r}\right)l_{r}^{2}\right)}{v_{x}I_{z}} \end{array}\right]$; $\text{B}=\left[\begin{array}{cc} \frac{2C_{f}\left(\varphi_{f}\right)}{mv_{x}} & 0\\ \frac{2C_{f}\left(\varphi_{f}\right)l_{f}}{I_{z}} & \frac{1}{I_{z}} \end{array}\right]$; **C** = [0, 1]; $M_{z}=\frac{d}{2}\left(F_{rr}^{x}-F_{rl}^{x}\right)+\frac{d}{2}\left(F_{fr}^{x}-F_{fl}^{x}\right)$ is yaw moment; *d* is track width, the front and rear track widths are assumed to be equal in this study; β is vehicle sideslip angle; γ is yaw rate; δ_*f*_ is front steering angle; *C*_*f*_(φ_*f*_) is front-tire cornering stiffness; *C*_*r*_(φ_*r*_) is rear-tire cornering stiffness; *m* is vehicle mass; *l*_*f*_ is front-axle distance; *l*_*r*_ is rear-axle distance; *I*_*z*_ is yaw moment of inertia; $F_{fl}^{x}$ is front-left tire longitudinal force; $F_{fr}^{x}$ is front-right tire longitudinal force; and $F_{rl}^{x}$ is rear-left tire longitudinal force; $F_{rr}^{x}$ is rear-right tire longitudinal force.

### 3.2. Sumi-uncertainty dynamic modeling

The stability region of vehicle motion can be simplified as follows (Lian et al., 2018):


(8) $$\begin{array}{l} |c_{1}\beta +c_{2}\dot{\beta }|<1 \end{array}$$

where *c*_1_ and *c*_2_ are constant coefficients. Referring to the equation (8), β≡0 can satisfy (8) (Tjonnaas and Johansen, 2006). Therefore, substituting $\overset{•}{\beta }=\beta =0$ and $\overset{•}{\gamma }=0$ into (7), the stability condition between yaw moment and front steering angle can be obtained as follows:


(9) $$\begin{array}{l} M_{z}\left(s\right)=\frac{2C_{f}\left(\varphi_{f}\right)l_{f}mv_{x}^{2}-4C_{f}\left(\varphi_{f}\right)C_{r}\left(\varphi_{r}\right)l_{r}\left(l_{f}+l_{r}\right)}{2\left(C_{r}\left(\varphi_{r}\right)l_{r}-C_{f}\left(\varphi_{f}\right)l_{f}\right)-mv_{x}^{2}}\delta_{f}\left(s\right) \end{array}$$

Substituting (9) into (7), two-input and one-output model can be transformed in to a single-input and single-output (SISO) model, which is a stable simplified model, as follows:


(10) $$\begin{array}{l} \{\begin{array}{l} \overset{.}{x}=\bar{A}x+\bar{B}\bar{u}\\ y=\bar{C}x \end{array} \end{array}$$

where, **x** = [β, γ]^*T*^; $\bar{\text{u}}=\delta_{f}$; **y** = γ; $\bar{\text{A}}=\left[\begin{array}{cc} -\frac{2\left(C_{f}\left(\varphi_{f}\right)+C_{r}\left(\varphi_{r}\right)\right)}{mv_{x}} & \frac{2\left(C_{r}\left(\varphi_{r}\right)l_{r}-C_{f}\left(\varphi_{f}\right)l_{f}\right)}{mv_{x}^{2}}-1\\ \frac{2\left(C_{r}\left(\varphi_{r}\right)l_{r}-C_{f}\left(\varphi_{f}\right)l_{f}\right)}{I_{z}} & -\frac{2\left(C_{f}\left(\varphi_{f}\right)l_{f}^{2}+C_{r}\left(\varphi_{r}\right)l_{r}^{2}\right)}{v_{x}I_{z}} \end{array}\right]$;

$\bar{\text{B}}=\left[\begin{array}{c} \frac{2C_{f}\left(\varphi_{f}\right)}{mv_{x}}\\ \frac{4C_{f}\left(\varphi_{f}\right)\left(C_{f}\left(\varphi_{f}\right)l_{f}^{2}+C_{r}\left(\varphi_{r}\right)l_{r}^{2}\right)}{\left(2C_{f}\left(\varphi_{f}\right)l_{f}-2C_{r}\left(\varphi_{r}\right)l_{r}+mv_{x}^{2}\right)I_{z}} \end{array}\right]$; $\bar{\text{C}}=\left[0,1\right]$.

Referring to (10), *m* and *I*_*z*_ are not known exactly in fact, and *C*_*f*_(φ_*f*_) and *C*_*r*_(φ_*r*_) are regarded as constants in this study, namely, *C*_*f*_(φ_*f*_) = *C*_*f*_, *C*_*r*_(φ_*r*_) = *C*_*r*_. Assuming that their values are represented within certain, known intervals, and *v*_*x*_ is constant in steering process. *m* and *I*_*z*_ can be described as upper linear fractional transformations (ULFT; Lian et al., 2018; Nie et al., 2023), respectively.


(11) $$\begin{array}{l} \{\begin{array}{l} m=\bar{m}\left(1+p_{m}\delta_{m}\right)=F_{u}\left(M_{m1},\delta_{m1}\right)\\ \frac{1}{m}=\frac{1}{\bar{m}}-\frac{p_{m}}{\bar{m}}\delta_{m}{\left(1+p_{m}\delta_{m}\right)}^{-1}=F_{u}\left(M_{m2},\delta_{m2}\right)\\ \frac{1}{I_{z}}=\frac{1}{{\bar{I}}_{z}}-\frac{p_{I_{z}}}{{\bar{I}}_{z}}\delta_{I_{z}}{\left(1+p_{I_{z}}\delta_{I_{z}}\right)}^{-1}=F_{u}\left(M_{\frac{1}{I_{z}}},\delta_{I_{z}}\right) \end{array} \end{array}$$

where, $\bar{m}$ and ${\bar{I}}_{z}$ are the nominal values of *m*, *I*_*z*_; *p*_*m*_, *p*_*I*_*z*__, δ_*m*_, and δ_*I*_*z*__ represent the relative perturbations on these parameters, 0 ≤ δ_*m*1_ ≤ 1, 0 ≤ δ_*m*2_ ≤ 1, 0 ≤ δ_*I*_*z*__ ≤ 1; ${\text{M}}_{m1}=\left[\begin{array}{cc} 0 & \bar{m}\\ p_{m} & \bar{m} \end{array}\right]$; ${\text{M}}_{m2}=\left[\begin{array}{cc} -p_{m} & \frac{1}{\bar{m}}\\ -p_{m} & \frac{1}{\bar{m}} \end{array}\right]$; ${\text{M}}_{\frac{1}{I_{z}}}=\left[\begin{array}{cc} -p_{I_{z}} & \frac{1}{{\bar{I}}_{z}}\\ -p_{I_{z}} & \frac{1}{{\bar{I}}_{z}} \end{array}\right]$.

**Figure 5:**
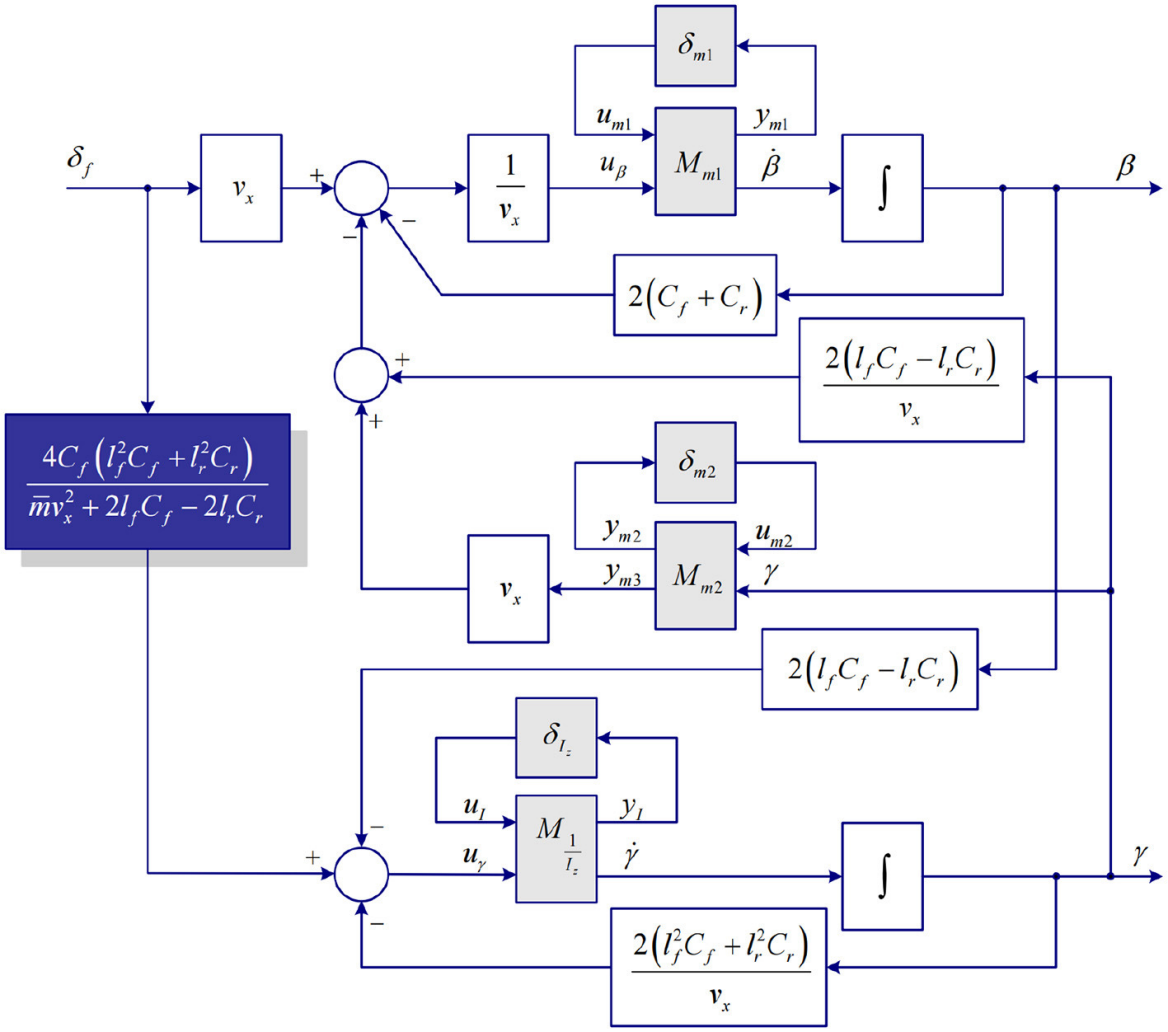
Vehicle semi-uncertainty dynamic model.

Combining with (11), a semi-uncertainty dynamic model (SUDM) shown in Figure 5 can be obtained. The model in the blue box on the left satisfies the condition of handling stability (8), and the uncertainty of parameters is not considered in this part of the model. The uncertainty of the vehicle parameters in the model can be represented by the model in the gray box on the right. The whole vehicle model is defined as sumi-uncertainty dynamic model (SUDM) in this study. Referring to Figure 5, the state space representation of input/output SUDM can be deduced as follows:


(12) $$\begin{array}{l} Y_{SUDM}=G_{SUDM}\cdot u_{SUDM}=\left[\begin{array}{ccc} \tilde{A} & {\tilde{B}}_{1} & {\tilde{B}}_{2}\\ {\tilde{C}}_{1} & {\tilde{D}}_{11} & {\tilde{D}}_{12}\\ {\tilde{C}}_{2} & {\tilde{D}}_{21} & {\tilde{D}}_{22} \end{array}\right]\cdot u_{SUDM} \end{array}$$

$\begin{array}{l} \text{where, }y_{SUDM}={\left[y_{m1},y_{m2},y_{I},y\right]}^{T};u_{SUDM}={\left[u_{m1},u_{m2},u_{I},u\right]}^{T};\\ \tilde{A}=\left[\begin{array}{cc} -\frac{2\left(C_{f}+C_{r}\right)}{\bar{m}v_{x}} & -\frac{2\left(C_{f}l_{f}-C_{r}l_{r}\right)}{\bar{m}v_{x}^{2}}-1\\ -\frac{2\left(C_{f}l_{f}-C_{r}l_{r}\right)}{{\bar{I}}_{z}} & -\frac{2\left(C_{f}l_{f}^{2}+C_{r}l_{r}^{2}\right)}{{\bar{I}}_{z}v_{x}} \end{array}\right];{\tilde{B}}_{1}=\left[\begin{array}{ccc} 1 & -\frac{1}{\bar{m}} & 0\\ 0 & 0 & 1 \end{array}\right];\\ {\tilde{B}}_{2}=\left[\begin{array}{c} \frac{2C_{f}}{\bar{m}v_{x}}\\ \frac{4C_{f}\left(C_{f}l_{f}^{2}+C_{r}l_{r}^{2}\right)}{{\bar{I}}_{z}\left(\bar{m}v_{x}^{2}+2C_{f}l_{f}-2C_{r}l_{r}\right)} \end{array}\right];\\ {\tilde{C}}_{1}=\left[\begin{array}{cc} \frac{2p_{m}\left(C_{f}+C_{r}\right)}{\bar{m}v_{x}} & \frac{2p_{m}\left(C_{f}l_{f}-C_{r}l_{r}\right)}{\bar{m}v_{x}^{2}}+p_{m}\\ 0 & p_{m}\bar{m}\\ \frac{2p_{I}\left(C_{f}l_{f}-C_{r}l_{r}\right)}{{\bar{I}}_{z}} & \frac{2p_{I}\left(C_{f}l_{f}^{2}+C_{r}l_{r}^{2}\right)}{v_{x}{\bar{I}}_{z}} \end{array}\right];{\tilde{C}}_{2}=\left[0,1\right]\text{; }\\ {\tilde{D}}_{11}=\left[\begin{array}{ccc} -p_{m} & \frac{p_{m}}{\bar{m}} & 0\\ 0 & 0 & 0\\ 0 & 0 & -p_{I} \end{array}\right];\\ {\tilde{D}}_{12}=\left[\begin{array}{c} -\frac{2p_{m}C_{f}}{\bar{m}v_{x}}\\ -\frac{4p_{I}C_{f}\left(C_{f}l_{f}^{2}+C_{r}l_{r}^{2}\right)}{{\bar{I}}_{z}\left(\bar{m}v_{x}^{2}+2C_{f}l_{f}^{2}-C_{r}l_{r}^{2}\right)} \end{array}\right];{\tilde{D}}_{21}=\left[0,0,0\right];{\tilde{D}}_{22}=\left[0\right]; \end{array}$ Unknown transfer function matrix $△=\left[\begin{array}{ccc} \delta_{m1} & 0 & 0\\ 0 & \delta_{m2} & 0\\ 0 & 0 & \delta_{I} \end{array}\right]$, which can represent vehicle dynamic perturbations and ||△||_∞_ ≤ 1.

### 3.3. Direct yaw-moment controller design

**Figure 6:**
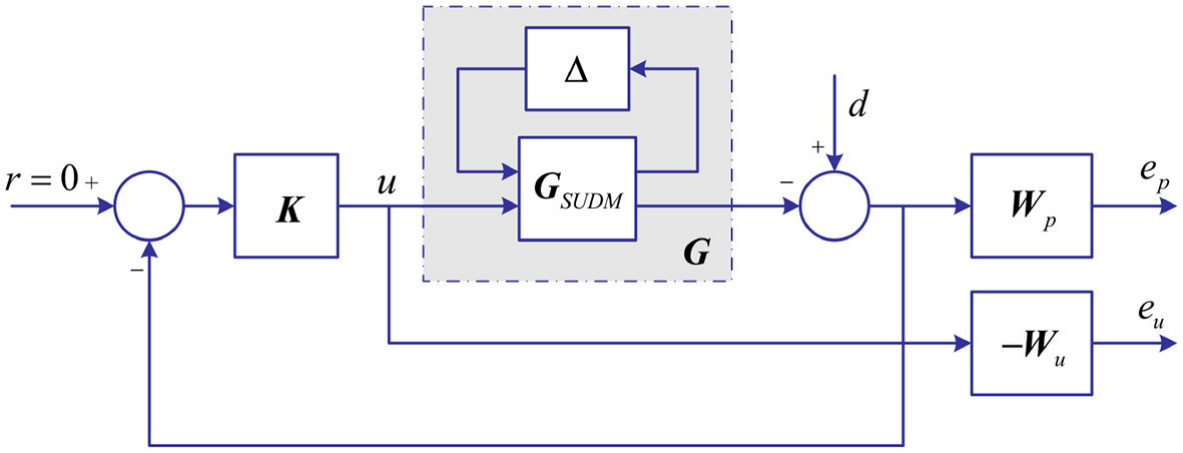
The closed-loop structure of vehicle DYC robust control system.

For the robust stability, the closed-loop system, for all **G** = *F*_*u*_(**G**_*SUDM*_, △), must satisfy the performance criterion, namely, S/KS mixed sensitivity problem,


(13) $$\begin{array}{l} ||\left[\begin{array}{c} W_{p}S\\ W_{u}KS \end{array}\right]|{|}_{\infty }=||\left[\begin{array}{c} W_{p}{\left(I+GK\right)}^{-1}\\ W_{u}K{\left(I+GK\right)}^{-1} \end{array}\right]|{|}_{\infty }<1 \end{array}$$

where **W**_*p*_ is used to represent the frequency characteristics of external disturbance; **W**_*u*_ is used to constrain control output to prevent the longitudinal force difference between the left and right tires from exceeding their limits determined by in-wheel-motor; **S** is defined as sensitivity function; **K** is robust controller.For the closed-loop structure of vehicle DYC robust controller shown in Figure 6, the relationship between input and output is given as follows:


(14) $$\begin{array}{l} \left[\begin{array}{c} e_{p}\\ e_{u}\\ y \end{array}\right]=\left[\begin{array}{cc} P_{11} & P_{12}\\ P_{21} & P_{22} \end{array}\right]\left[\begin{array}{c} d\\ u \end{array}\right] \end{array}$$

where, ${\text{P}}_{11}=\left[\begin{array}{c} {\text{W}}_{p}\\ \text{0} \end{array}\right]$; ${\text{P}}_{12}=\left[\begin{array}{c} -{\text{W}}_{p}\text{G}\\ {\text{W}}_{u} \end{array}\right]$; **P**_21_ = **I**; **P**_22_ = −**G**.

The transfer functions from *d* to $e={\left[e_{p},e_{u}\right]}^{T}$ can be obtained as follows:


(15) $$\begin{array}{l} F_{l}\left(\mathrm{P,K}\right)=P_{11}+P_{12}K{\left(I-P_{22}K\right)}^{-1}P_{21}=\left[\begin{array}{c} W_{p}{\left(I+GK\right)}^{-1}\\ W_{u}K{\left(I+GK\right)}^{-1} \end{array}\right] \end{array}$$

Therefore, the S/KS mixed sensitivity problem (13) based on SUDM can be transformed into *H*_∞_ standard problem (16) based on SUDM, namely,


(16) $$\begin{array}{l} ||F_{l}\left(\mathrm{P,K}\right)|{|}_{\infty }<1 \end{array}$$

## 4. Simulation and analysis

### 4.1. DYC control system simulation platform

**Figure 7:**
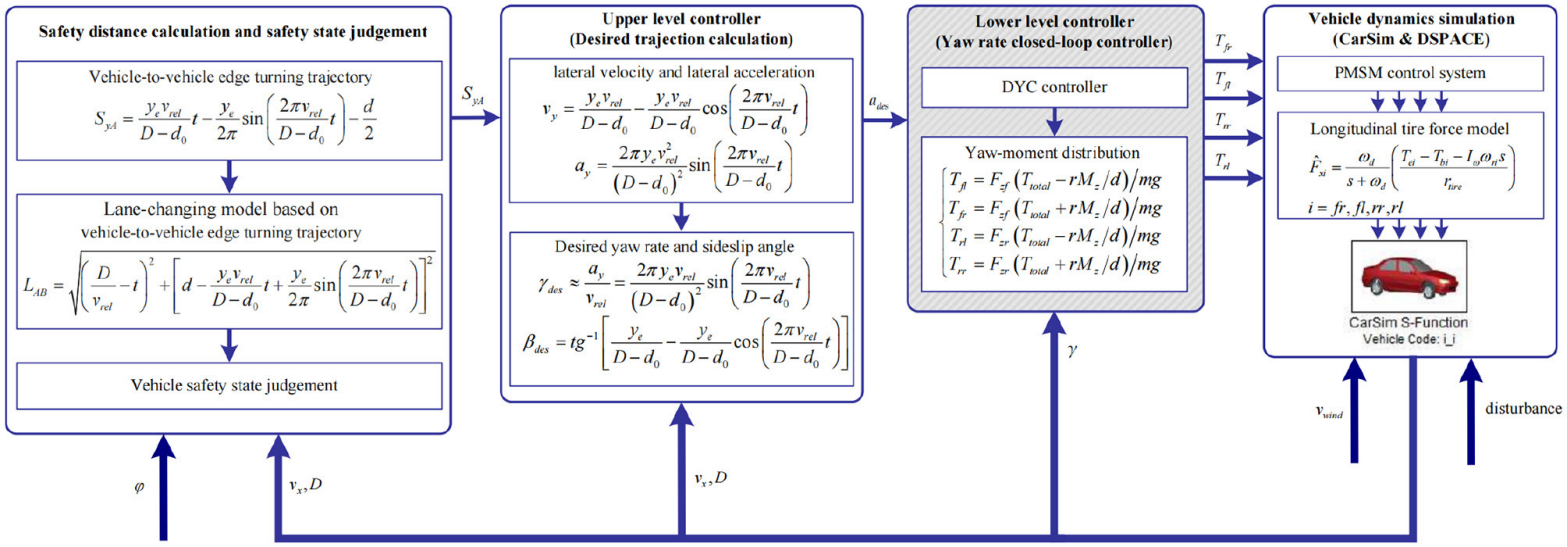
Structure diagram of the DYC control system. *d*_0_ is longitudinal minimum distance. *v*_*rel*_ is relative velocity, *v*_*rel*_ = *v*_*x*_ with leading vehicle velocity is 0. *y*_*e*_ is lane width. *D* is vehicle-to-vehicle distance. Disturbance is white noise.

Structural drawing of DYC control system of electric vehicles is shown in Figure 7, which is the control system structure of the following vehicle. The leading vehicle is regarded as stopping in front of the following vehicle. The following vehicle completes the lane change to avoid collision. Based on authors' previous studies (Lian et al., 2018), safety distance calculation, vehicle safety state judgement, hierarchical controllers, and yaw-moment distribution are used again in this study.

### 4.2. Adhesion coefficient and cornering stiffness normalization

#### 4.2.1. Data acquisition and FOMVGM modeling

**Figure 8:**
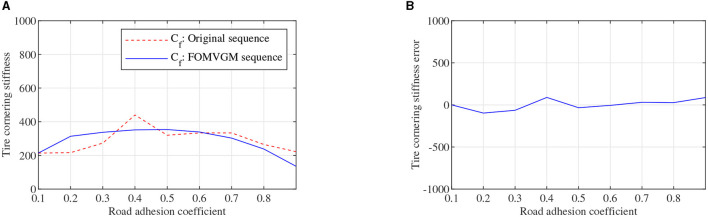
Tire cornering stiffness of the front tire, **(A)** is original sequence and FOMVGM sequence, **(B)** is the error between original sequence and FOMVGM sequence.

**Figure 9:**
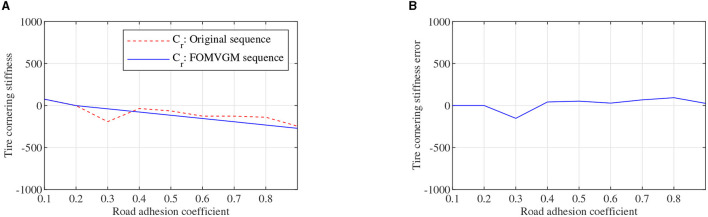
Tire cornering stiffness of the rear tire, **(A)** is original sequence and FOMVGM sequence, **(B)** is the error between original sequence and FOMVGM sequence.

To obtain the feasible and effective data to build the FOMVGM between road adhesion coefficient and tire cornering stiffness, the data on tire lateral force and tire sideslip angle are extracted from CarSim under different road friction coefficient peak values (Li et al., 2016). Tire cornering stiffnesses of front and rear tires can be fitted with the Magic Formula tire model as the original sequences. Original sequences of tire cornering stiffnesses are all sequences of nine elements corresponding to nine different adhesion coefficients. As shown in Figures 8A, 9A, tire cornering stiffnesses of the front and rear tires can be fitted by the FOMVGM with fractional order *r* = 0.015, respectively, and the tire cornering stiffness errors of the front and rear tires are also presented in Figures 8B, 9B. By FOMVGM calculation, the mean relative percentage error (MRPE) of the front tire cornering stiffness is 17.7 <20%, and the MRPE of the rear tire cornering stiffness is 33.79 <50%. It can demonstrate that the FOMVGM has good data fitting accuracy and is suitable for fitting tire cornering stiffness. Therefore, FOMVGM could generate training data and testing data for the LSTM network.

#### 4.2.2. Normalization based on LSTM network

**Figure 10:**
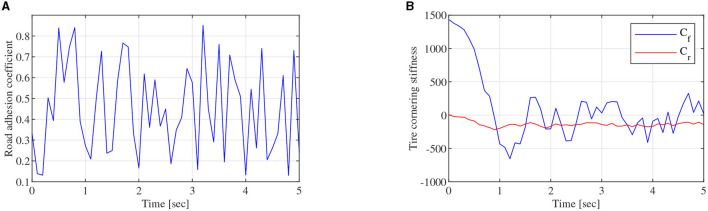
Training data generation, **(A)** is random data between 0.1 and 0.9, **(B)** is tire cornering stiffness data of the front and tires obtained by the FOMVGM.

**Figure 11:**
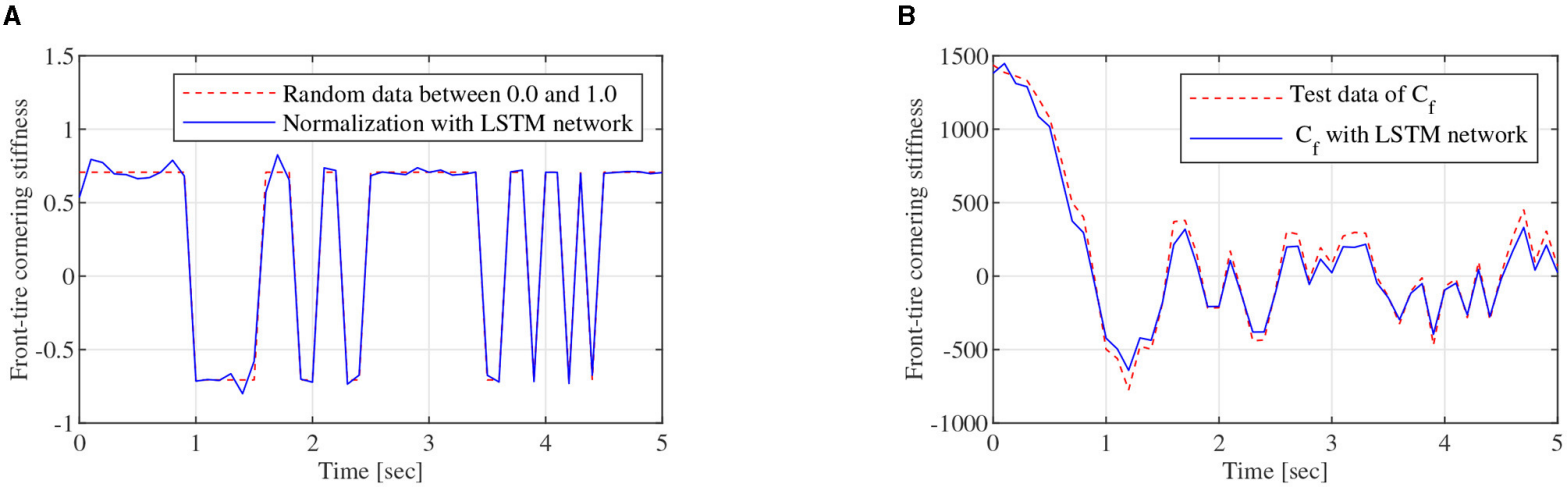
Training data generation, **(A)** is the normalization data of *C*_*f*_, **(B)** is tire cornering stiffness calculating data of *C*_*f*_ obtained by the LSTM network.

**Figure 12:**
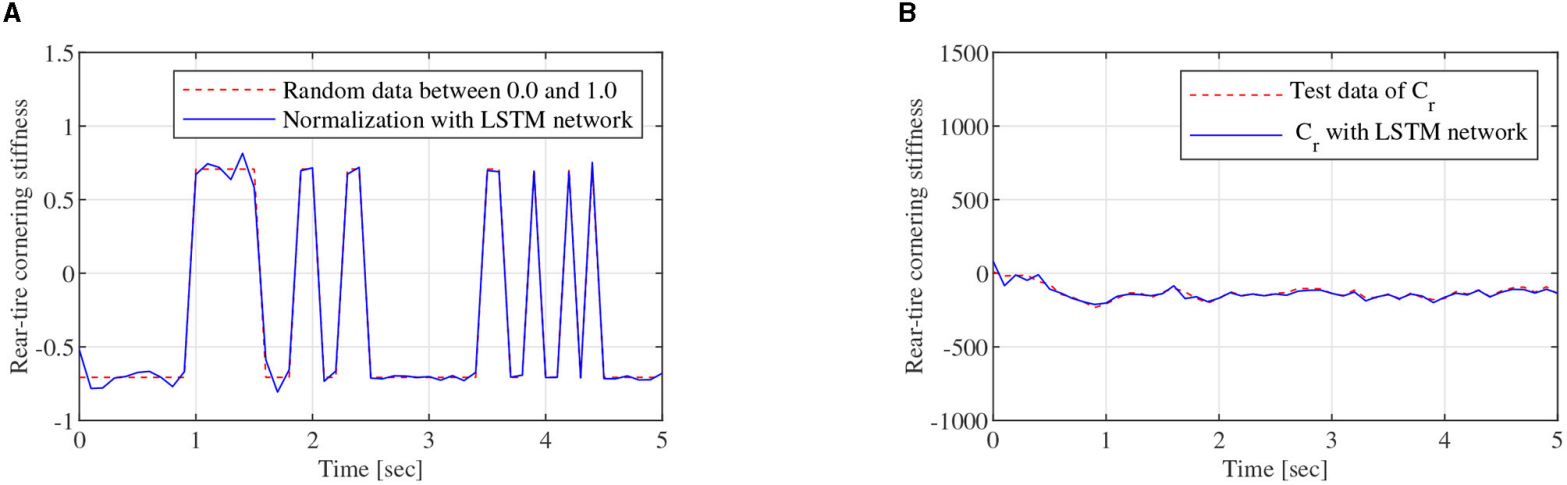
Training data generation, **(A)** is the normalization data of *C*_*r*_, **(B)** is tire cornering stiffness calculating data of *C*_*r*_ obtained by the LSTM network.

As shown in Figure 3, the regression model of the LSTM network, which is constructed with two LSTM layers, a full connected layer, and a regression layer, is a two-node input and two-node out network. To train the LSTM network, the FOMVGM built in Section 2 is used to calculate training data and test data. As shown in Figure 10A, random data between 0.1 and 0.9, which can represent the different road adhesion coefficients, are used as the input of the FOMVGM. As shown in Figure 10B, the tire cornering stiffness can be calculated by the FOMVGM. Input data (road adhesion coefficient) are extracted by 200 points, and output data (front-tire cornering stiffness and rear-tire cornering stiffness) are also extracted by 200 points, respectively. The data provide the training data for the supervised learning of LSTM network. In the LSTM network, the input layer has one node and output layer has two nodes. Each layer of the LSTM has 20 neurons. The LSTM network iteration is set to 300, the initial learning rate is 0.005, and the final learning rate is 0.00004. Mini-batch RMSE is 0.00934. Elapsed time is 23 s. Training effect of LSTM network is good. Random data between 0.0 and 1.0 are employed to test the LSTM network. As shown in Figures 11A, 12A, the normalization data of the front-tire and rear-tire cornering stiffness can be shown with different road adhesion coefficients. As shown in Figures 11B, 12B, tire cornering stiffness of the front and rear tires can be well-obtained with LSTM network computation, which can participate in vehicle dynamics system modeling and DYC robust controller design.

### 4.3. DYC robust control simulation

Simulation experiments with CarSim are conducted with tire cornering stiffness calculated by the LSTM network. Electric vehicle and robust controller parameters used in this study are presented in Table 1. In simulation experiments, road adhesion coefficient can be set with different values to verify the feasibility and the effectiveness of the DYC control system in different driving cycles, namely, single driving cycle and mixed driving cycle. In addition to that, a virtual leading vehicle is presented to imitate lane changing condition of the following vehicle. The virtual leading vehicle can be described with vehicle-to-vehicle distance *D*_0_, and the longitudinal velocity of the virtual leading vehicle is 0 m/s. In this study, *D*_0_ is set to 120 m, and *v*_*x*_ is set to 90 kph.

**Table 1:** Vehicle dynamics and robust controller parameters.

**Parameter**	**Value**	**Parameter**	**Value**	**Parameter**	**Value**	**Parameter**	**Value**
$\bar{m}$	1,159 kg	*v*_*x*_	90 kph	*l*_*f*_	1.04 m	*l*_*r*_	1.56 m
*d*_0_	$10\cdot \frac{2}{\varphi +0.3}$	*r*	0.313 m	***W***_*p*_	$\frac{0.095s^{2}+15.01s+9.5}{s^{2}+0.5s+0.005}$	***W***_*u*_	10^−2^
*p*_*m*_	0.2	*p*_*I*_	0.3	${\bar{I}}_{z}$	617 kg·*m*^2^	*D*_0_^a^	120 m

^a^*D*_0_ is initial vehicle-to-vehicle distance.

#### 4.3.1. Single driving cycle simulation

**Figure 13:**
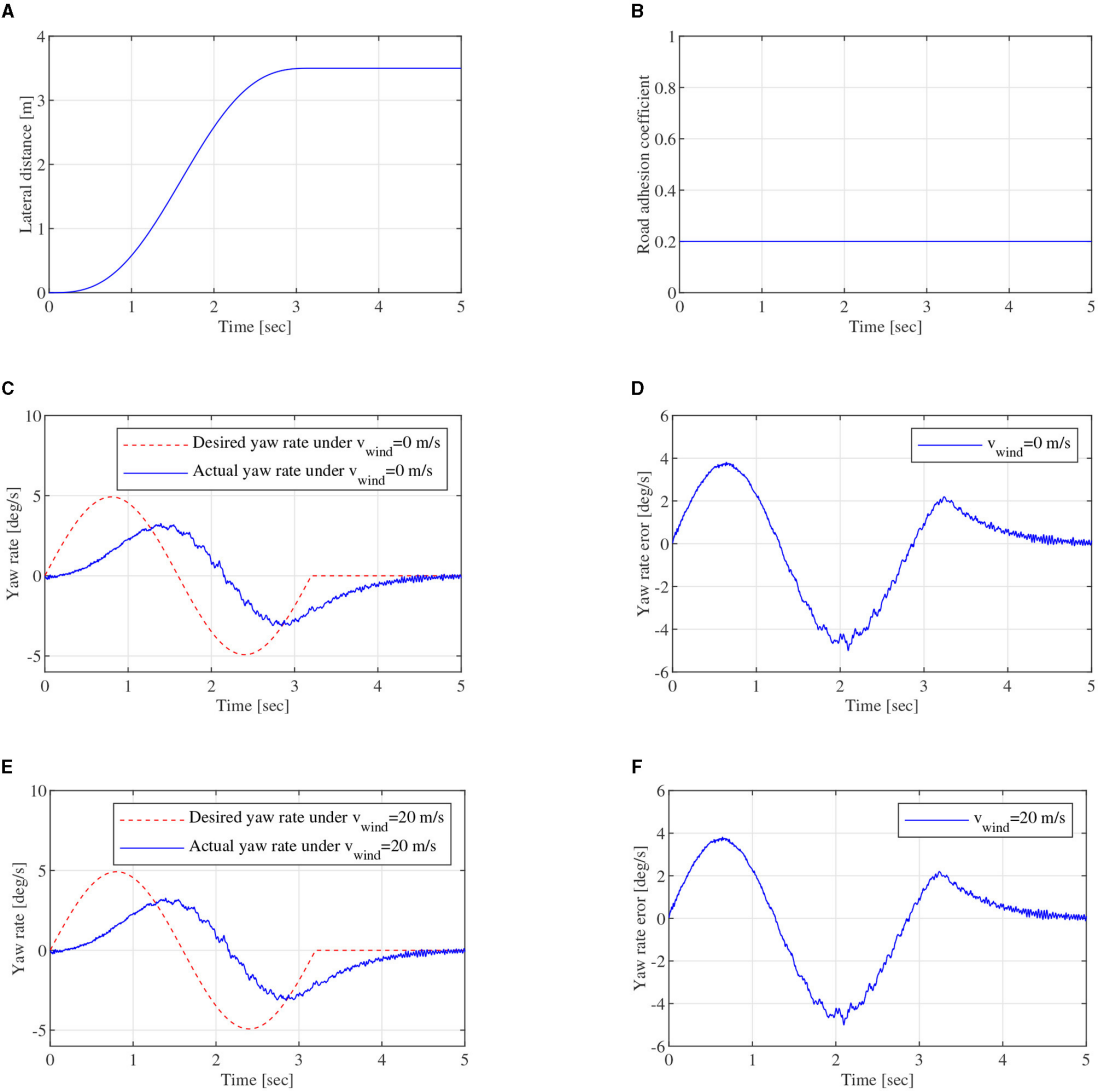
Single driving cycle, **(A)** is lateral distance, **(B)** is road adhesion coefficient of the single driving cycle, **(C)** is yaw rate close-loop control curve of DYC robust control under *v*_*wind*_ = 0*m*/*s*, **(D)** is yaw rate error under *v*_*wind*_ = 0*m*/*s*, **(E)** is yaw rate close-loop control curve of DYC robust control under *v*_*wind*_ = 20*m*/*s*, **(F)** is yaw rate error under *v*_*wind*_ = 20*m*/*s*.

In single driving cycle simulation, vehicle-to-vehicle edge turning trajectory, which can be expressed by the formula in Figure 7, can be calculated and shown in Figure 13A. φ is set to 0.2, which is shown in Figure 13B. Furthermore, *d*_0_ = 40*m*. Lateral wind velocity *v*_*wind*_ and white noise can be used as driving environment disturbance. Figures 13C, E show yaw rate closed-loop robust control effect under *v*_*wind*_ = 0 and *v*_*wind*_ = 20*m*/*s*, respectively. Actual yaw rate could track desired yaw rate, though there are some errors in the tracking process. The errors are mainly caused by the vehicle inertia *I*_*z*_ change and large longitudinal velocity *v*_*x*_. Due to vehicle inertia, actual yaw rate can not follow the desired yaw rate immediately, when desired yaw rate fluctuates. Figures 13D, F show the absolute error curves under *v*_*wind*_ = 0 and *v*_*wind*_ = 20*m*/*s*, respectively. The error ranges of yaw rate are all between –5.00 and 3.81 *deg*/*s*. With the robust controller, the yaw rate error is reduced quickly. It can demonstrate that the designed robust controller based on SUDM can overcome lateral wind disturbance from *v*_*wind*_ = 0 to *v*_*wind*_ = 20*m*/*s* besides vehicle parameters perturbation. The feasibility and effectiveness of DYC control system can be verified in single driving cycle.

#### 4.3.2. Mixed driving cycle simulation

**Figure 14:**
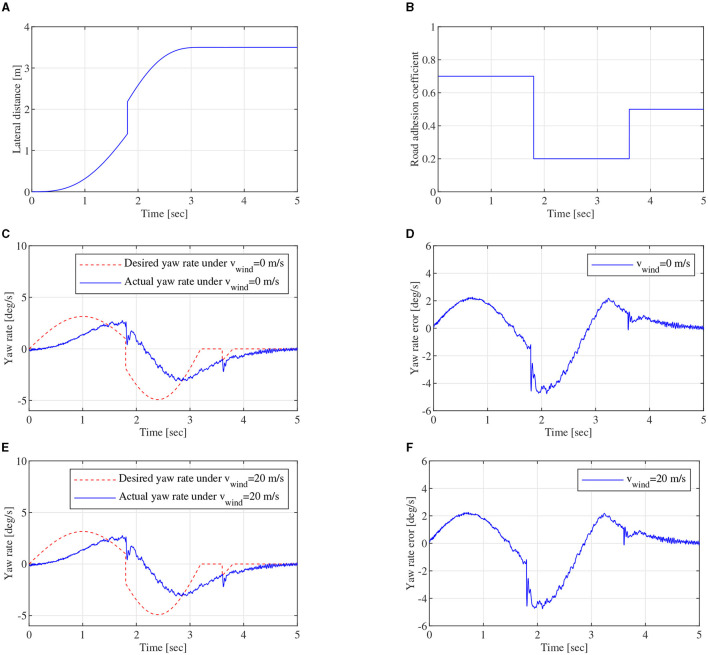
Mixed driving cycle, **(A)** is lateral distance, **(B)** is road adhesion coefficient of the mixed driving cycle, **(C)** is yaw rate close-loop control curve of DYC robust control under *v*_*wind*_ = 0*m*/*s*, **(D)** is yaw rate error under *v*_*wind*_ = 0*m*/*s*, **(E)** is yaw rate close-loop control curve of DYC robust control under *v*_*wind*_ = 20*m*/*s*, **(F)** is yaw rate error under *v*_*wind*_ = 20*m*/*s*.

To further validate the robustness performance of DYC control system for different driving cycles, mixed driving cycle simulation experiments can be carried out. In mixed driving cycle simulation, vehicle-to-vehicle edge turning trajectory, which can be expressed by the formula in Figure 7, can be calculated and shown in Figure 14A. As shown in Figure 14B, φ is a piecewise function, which contains the values 0.7, 0.2, and 0.5. The piecewise function can represent complex pavement in this study. Lateral wind velocity *v*_*wind*_ and white noise can be still used as driving environment disturbance. Figures 14C, E show yaw rate closed-loop robust control effect under *v*_*wind*_ = 0 and *v*_*wind*_ = 20*m*/*s*, respectively. The desired yaw rate fluctuates at ~1.8 and 3.7 s, and the actual yaw rate also fluctuates at ~1.8 and 3.7 s in the tracking process under *v*_*wind*_ = 0 and *v*_*wind*_ = 20*m*/*s*. The fluctuation phenomenon is mainly caused by the change in the different adhesion coefficients. Figures 14D, F show the absolute error curves under *v*_*wind*_ = 0 and *v*_*wind*_ = 20*m*/*s*, respectively. The error ranges of yaw rate are all between –4.76 and 2.26 *deg*/*s*. With the robust controller, the yaw rate error can be also reduced quickly. It can demonstrate that the designed robust controller based on SUDM can overcome lateral wind disturbance from *v*_*wind*_ = 0 to *v*_*wind*_ = 20*m*/*s* besides vehicle parameters perturbation. The feasibility and effectiveness of DYC control system can be verified in mixed driving cycle.

## 5. Conclusion

This study proposes a normalization method-based LSTM network of road adhesion coefficient and tire cornering stiffness, to provide the significant information for vehicle DYC control system. A FOMVGM is built to generate numerous data for training and testing the LSTM network. Tire cornering stiffness can be estimated well by the LSTM network with road adhesion coefficient. In addition to that, a DYC control system-based SUDM is presented. The feasibility and effectiveness of the DYC control system are verified by single driving cycle and mixed driving cycle. An SUDM can not only ensure the stable steering of vehicles but also achieve good control effect with single variable, yaw rate, instead of double variables, yaw rate, and sideslip angle. The constrained problem between the direct measurement cost of sideslip angle and the joint control of yaw rate and sideslip angle can be solved well.

## Data availability statement

The original contributions presented in the study are included in the article/supplementary material, further inquiries can be directed to the corresponding author.

## Author contributions

YL and ZN: data curation, writing—original draft, validation, and supervision. YL and SL: conceptualization. WF, SL, and ZN: methodology. YL and WF: software. YL, SL, and ZN: formal analysis. All authors have read and agreed to the published version of the manuscript.

## References

[B1] Alonso J. López J. Pavón I. Asensio C. Areas G. (2015). “Platform for on-board real-time detection of wet, icy and snowy roads, using tyre/road noise analysis,” in 2015 International Symposium on Consumer Electronics (ISCE), (Madrid), 1–2.

[B2] Breuer B. Eichhorn U. Roth J. (1992). Measurement of Tyre/Road-Friction ahead of the car and inside the tyre. Proc. AVEC. 347–353.

[B3] Ding X. Wang Z. Zhang L. Wang C. (2020). Longitudinal vehicle speed estimation for four-wheel-independently-actuated electric vehicles based on multi-sensor fusion. IEEE Trans. Vehicul. Technol. 69, 12797–12806. 10.1109/TVT.2020.3026106

[B4] Douangphachanh V. Oneyama H. (2014). “Formulation of a simple model to estimate road surface roughness condition from android smartphone sensors,” in 2014 IEEE Ninth International Conference on Intelligent Sensors, Sensor Networks and Information Processing (ISSNIP) (Singapore), 1–6.

[B5] Hahn J.-O. Rajamani R. Alexander L. (2002). GPS-based real-time identification of tire-road friction coefficient. IEEE Trans. Contr. Syst. Technol. 10, 331–343. 10.1109/87.998016

[B6] Han K. Choi M. Choi S. B. (2018). Estimation of the tire cornering stiffness as a road surface classification indicator using understeering characteristics. IEEE Trans. Vehicul. Technol. 67, 6851–6860. 10.1109/TVT.2018.2820094

[B7] Han K. Lee E. Choi M. Choi S. B. (2017). Adaptive scheme for the real-time estimation of tire-road friction coefficient and vehicle velocity. IEEE/ASME Trans. Mechatr. 22, 1508–1518. 10.1109/TMECH.2017.2704606

[B8] Junmin W. Lee A. Rajesh R. (2004). Fricition estimation on highway vehicles using longitudinal measurement. J. Dyn. Syst. Measur. Contr. 126, 265–275. 10.1115/1.1766028

[B9] Lee C. Hedrick K. Yi K. (2004). Real-time slip-based estimation of maximum tire-road friction coefficient. IEEE/ASME Trans. Mechatr. 9, 454–458. 10.1109/TMECH.2004.828622

[B10] Li L. Ma L. Mu Y. Xu C. Li W. Shi S. (2016). Parameter identification method for the tire cornering stiffness of model vehicle. Automot. Eng. 38, 1508–1514. 10.1109/CDC.2005.1583244

[B11] Li S. Yang Z. (2022). “AFS/DYC control of in-wheel motor drive electric vehicle with adaptive tire cornering stiffness,” in 2022 6th CAA International Conference on Vehicular Control and Intelligence (CVCI) (Nanjing), 1–6.

[B12] Lian Y. Huang J. Liu S. Sun Z. Li B. Nie Z. (2022). Driving intention inference based on a deep neural network with dropout regularization from adhesion coefficients in active collision avoidance control. Electronics 11, 2284. 10.3390/electronics11152284

[B13] Lian Y. Wang X. Tian Y. Liu K. (2018). Lateral collision avoidance robust control of electric vehicles combining a lane-changing model based on vehicle edge turning trajectory and a vehicle semi-uncertainty dynamic model. Int. J. Automot. Technol. 19, 331–343. 10.1007/s12239-018-0032-1

[B14] Lian Y. Zhao Y. Hu L. Tian Y. (2015). Cornering stiffness and sideslip angle estimation based on simplified lateral dynamic models for four-in-wheel-motor-driven electric vehicles with lateral tire force information. Int. J. Automot. Technol. 16, 669–683. 10.1007/s12239-015-0068-4

[B15] Müller S. Uchanski M. Hedrick K. (2003). Estimation of the maximum tire-road friction coefficient. ASME J. Dyn. Syst. Measur. Contr. 108, 327–330. 10.1115/1.1636773

[B16] Nam K. Fujimoto H. Hori Y. (2012). Lateral stability control of in-wheel-motor-driven electric vehicles based on sideslip angle estimation using lateral tire force sensors. IEEE Trans. Vehicul. Technol. 61, 1972–1985. 10.1109/TVT.2012.2191627

[B17] Nie Z. Wang C. Wang W. Zhao W. Lian Y. Chen H. (2023). Robust control of lateral obstacle avoidance for intelligent vehicle shared-driven by people and vehicles based on dynamic early warning. Proc. Inst. Mech. Eng. 237, 895–912. 10.1177/09544070221085359

[B18] Shi J. He Q. Wang Z. (2021). A transfer learning LSTM network-based severity evaluation for intermittent faults of an electrical connector. IEEE Trans. Comp. Packag. Manufact. Technol. 11, 71–82. 10.1109/TCPMT.2020.3043011

[B19] Tian Y. Lian Y. Tian C. Sun Z. Liu K. (2019). “Sideslip angle and tire cornering stiffness estimations for four-in-wheel-motor-driven electric vehicles,” in 2019 Chinese Control Conference (CCC) (Guangzhou), 2418–2423.

[B20] Tjonnaas J. Johansen T. A. (2006). “Adaptive optimizing dynamic control allocation algorithm for yaw stabilization of an automotive vehicle using brakes,” in 2006 14th Mediterranean Conference on Control and Automation (Ancona), 1–6.

[B21] Wang Y. Geng K. Xu L. Ren Y. Dong H. Yin G. (2022). Estimation of sideslip angle and tire cornering stiffness using fuzzy adaptive robust cubature kalman filter. IEEE Trans. Syst. Man Cybernet. 52, 1451–1462. 10.1109/TSMC.2020.3020562

[B22] Wu Y. Li G. Fan D. (2021). Joint estimation of driving state and road adhesion coefficient for distributed drive electric vehicle. IEEE Access 9, 75460–75469. 10.1109/ACCESS.2021.3081443

[B23] Yuan Z. Zhang X. Liu S. Han X. Du Y. (2015). “Laser line recognition for autonomous road roughness measurement,” in 2015 IEEE International Conference on Cyber Technology in Automation, Control, and Intelligent Systems (CYBER) (Shenyang), 436–440.

[B24] Zhang L. Guo P. Wang Z. Ding X. (2022). An enabling tire-road friction estimation method for four-in-wheel-motor-drive electric vehicles. IEEE Trans. Transport. Electrif. 2022, 1. 10.1109/TTE.2022.3231707

